# Patient autonomy in advance care planning for Parkinson’s disease: Systematic review with narrative synthesis

**DOI:** 10.1017/S1478951526103277

**Published:** 2026-07-23

**Authors:** Evie Z. Bouma, Jan W. Schoones, Ad A. Kaptein, Barend W. Florijn

**Affiliations:** 1 Neurology, Leiden University Medical Center, Netherlands; 2 Directorate of Research Policy, Leiden University Medical Center, Netherlands; 3 Medical Psychology, Leiden University Medical Center, Netherlands

**Keywords:** Autonomy, Parkinson’s disease, advance care planning, palliative care planning, narrative medicine

## Abstract

**Objectives:**

Individuals with Parkinson’s disease (PD) often experience delays in initiating advance care planning (ACP) and palliative care (PC) which can impact their autonomy as cognitive function declines. Because patient autonomy and decision-making in ACP and PC for individuals with PD is insufficiently explored, this systematic review with narrative synthesis aims to fill this gap by reviewing the existing literature on these topics. It examines how awareness and timing influences ACP and PC implementation, how illness narratives shape decision-making and autonomy perceptions and the roles of caregivers and neurologists in supporting patient autonomy.

**Methods:**

We conducted a systematic review with narrative synthesis of original research investigating ACP and PC in individuals with PD in accordance with PRISMA guidelines. The methodological quality of included studies was assessed using the Critical Appraisal Skills Programme checklists. Illness narrative types were categorized according to Arthur W. Frank’s framework (chaos, restitution and quest narratives). Findings were synthesized narratively and organized thematically in line with the review objectives.

**Results:**

We included 42 studies using quantitative and qualitative methodologies involving 4154 individuals with PD and 2191 caregivers. Several themes were identified. Limited knowledge of ACP and PC explained its lower occurrence. ACP is often delayed due to uncertainty and misconceptions leading to late or crisis-driven discussions. Narrative medicine shows most individuals with PD share chaos narratives reflecting a focus on motor symptoms over autonomy. Lastly, individuals with PD expressed a need for greater autonomy support while ACP counseling is well received and improves understanding of care needs and end-of-life decisions.

**Significance of results:**

Delays in initiating ACP and PC are associated with diminished autonomy in individuals with PD. Narrative medicine could help neurologists start ACP and PC discussions earlier, supporting autonomy and thereby aligning care with patient preferences.

## Introduction

Parkinson’s disease (PD) is the most rapidly growing neurological disorder worldwide (Dorsey and Bloem 2018), with risk increasing with age and higher incidence, prevalence and mortality in men (Ben-Shlomo et al. 2024). Given its neurodegenerative nature, PD is associated with a 2.5–6-fold increased risk of dementia, while around 20% of patients already show cognitive impairment at diagnosis (Oikonomou et al. 2026). This can lead to alterations in essential decision-making abilities (Karlawish et al. 2013), thereby making it challenging for individuals with PD to envision their future selves (de Vito et al. 2012; Ernst et al. 2017). These changes highlight the importance of timely discussions when individuals with PD still have sufficient competency and personal autonomy in shaping their future care preferences.

Recognizing future care preferences in advance can be facilitated through advance care planning (ACP). Among the 5 key objectives of ACP, 2 primary goals in ACP-mediated decision-making are respecting individual patient autonomy and enhancing the quality of (clinical) relationships (Fleuren et al. 2020). We previously demonstrated that individuals with PD often rely on clinicians for decision-making, due to an inherent knowledge imbalance, creating a responsibility for healthcare providers to respect and promote autonomy (Florijn et al. 2023, 2024). Neurologists can support this autonomy through early implementation of ACP or PC (Childress 2017). Although individuals with PD stress the importance of early ACP discussions (Kundrick et al. 2023) they often encounter a lack of such discussions (Boersma et al. 2016; Seshadri et al. 2023) as neurologists tend to postpone these conversations until significant cognitive decline (Kurpershoek et al. 2021). Consequently, several significant challenges in ACP for individuals with PD have been highlighted, including the challenge of introducing ACP before the onset of cognitive impairment without jeopardizing the therapeutic relationship (Sokol et al. 2019). Therefore, failing to address ACP or palliative care planning for individuals with PD as the disease progresses could undermine a practice of respecting and promoting autonomy.

There is recent suggestion that narrative medicine in patients with PD could support advance or PC planning and interventions (Trahair and Mantri 2023). This approach integrates patient and family narratives to create decision-making tools. We previously demonstrated that PD metaphors in patient narratives, classified based on Arthur W. Frank’s illness narrative types namely restitution-stories, chaos-stories, and quest-stories (Frank 2013) emphasize stigmatization, thereby affecting patients’ sense of autonomy (Florijn et al. 2023). However, it remains unclear which narratives individuals with PD use to highlight the impact of their diagnosis, how they underscore the need for ACP or PC and whether it affects their sense of autonomy in care. Therefore, the objective of this systematic review with narrative synthesis was 3-fold. First, to examine if awareness of ACP or PC among individuals with PD influences its implementation. Second, to determine whether prioritizing motor symptom treatment affects the timing of ACP or PC discussions by neurologists. Lastly, to explore how specific illness narrative types impact decision-making and support for autonomy in individuals with PD.

## Methods

### Search of literature

A systematic literature search was conducted in PubMed, EMBASE, Web of Science, Cochrane Library, CINAHL, and PsycINFO using various search terms for ACP, PC, and PD (search strategy can be found in Supplemental File 1). The search was performed on February 2, 2026. We identified 377 published papers. In PubMed 114 results, in Embase 85 results, in Web of Science 80 results, in Cochrane Library 19 results including 3 trial register items, in CINAHL 49 results and in PsycINFO 30 results. Additional studies were identified by screening bibliographies of relevant review studies. The systematic review was not registered and does not require ethical approval as the data are already published and available.

### Inclusion and exclusion criteria

This study was conducted as a systematic review with narrative synthesis. We included original research articles that investigated ACP or PC in individuals with PD. Eligible study designs included qualitative studies, observational prospective studies, descriptive studies, cross-sectional studies, case studies, and cohort studies. Studies focusing exclusively on healthcare professionals’ experiences (e.g., neurologists’ perspectives without patient data) were excluded. Additional inclusion criteria were availability of the full text and publication in English. No restrictions were applied regarding outcome measures, allowing inclusion of studies reporting experiential, descriptive, or effectiveness-related findings relevant to ACP or PC in PD.

### Procedure for selection

EZB and BWF first screened articles based on their titles and abstracts, followed by a thorough examination of the full text. E.Z.B. and B.W.F. independently conducted this process and then reviewed each other’s work to ensure the validity and reliability of the selection procedure. In instances of disagreement, a third reviewer (A.A.K.) resolved any discrepancies. Studies meeting all eligibility criteria were included in the final review.

### Quality assessment

To independently assess the quality of all included papers, we used the Critical Appraisal Skills Programme (CASP) checklists, fitted to the individual study design, i.e., CASP qualitative-, CASP cohort-, or CASP case–control study (Programme 2024). In instances of disagreement, the third reviewer resolved any discrepancies. This quality assessment method is consistent with prior systematic reviews examining experiences of ACP in PD and atypical parkinsonian disorders (Nimmons et al. 2020).

### Extraction and synthesis of the data

A standardized data extraction form was developed to ensure consistency across studies. This data extraction form captured bibliographic and methodological characteristics (author, year, and study design), participant characteristics (number of individuals with PD, number of non-PD participants, and number of caregivers) and study aims. In addition, in alignment with the objectives of this review, data were extracted on:

1. Awareness and knowledge of ACP or PC among individuals with PD and whether this was associated with implementation or engagement in ACP or PC.
2. Timing of ACP or PC discussions, particularly in relation to disease stage.
3. Perceived autonomy support from healthcare professionals (neurologists, general practitioners, and physicians) and caregivers during ACP or PC discussions.
4. Caregiver involvement including their role in surrogate decision-making and participation in ACP or PC discussions.
5. Illness narrative type which was categorized according to Arthur W. Frank’s framework (chaos, restitution, and quest narratives) when inferable from qualitative findings (Frank 2013).
6. Effectiveness of interventions designed to enhance ACP and PC integration within clinical practice for patients with PD.

Given the heterogeneity of study designs, outcome measures, and reporting formats we used a narrative synthesis approach. Findings were summarized descriptively and organized thematically in accordance with the review objectives. The narrative synthesis focused on: (i) identifying associations between awareness of ACP or PC and its implementation, (ii) examining whether emphasis on motor symptom treatment influenced the timing of ACP or PC discussions and (iii) exploring how different illness narrative types shaped decision-making processes and perceptions of autonomy support. Recurring patterns, similarities, and differences across studies were systematically compared to develop an integrated interpretation of experiences, practices, and outcomes related to ACP and PC in PD.

### Narrative synthesis of the results

Following data extraction, we categorized the findings into these themes. 1. Knowledge of ACP or PC in individuals with PD. 2. Timing considerations for ACP or PC. 3. Perception of autonomy support in ACP or PC. 4. Caregivers’ involvement in surrogate decision-making concerning ACP or PC. 5. Narrative Medicine in ACP or PC. 6. Effectiveness of PC interventions.

## Results

### Description and characteristics of studies

**Figure 1.**
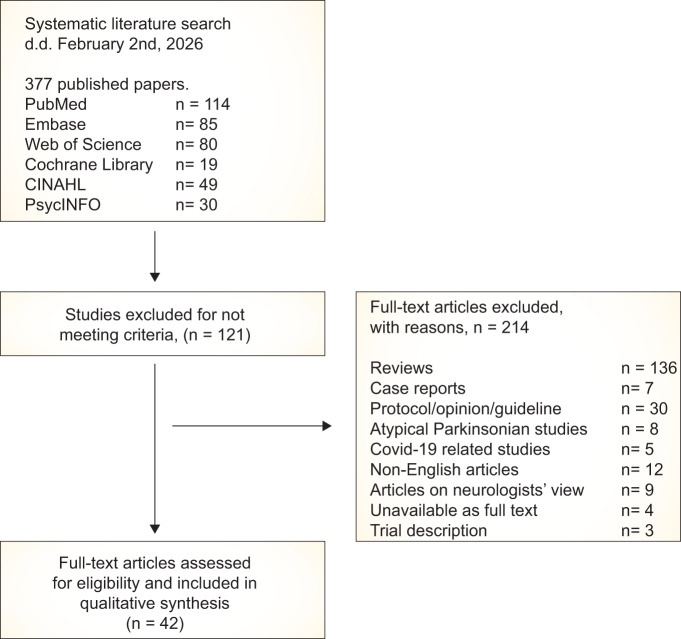
Article flowchart showing inclusions and exclusions. Figure 1 long description.

We initially identified 377 studies (Figure 1). Subsequent screening by title and abstract led to the exclusion of 121 articles that were irrelevant to the research question. Additionally, we excluded 136 review articles, 7 case reports, 30 protocol/expert-opinion/guideline articles, 9 survey articles among neurologists, 12 non-English articles, 4 articles unavailable as full text, 8 studies on atypical Parkinsonism, 3 trial descriptions and 5 articles related to COVID-19 PC. Finally, 42 articles were included. The included studies comprised 23 qualitative studies, 2 randomized controlled trials (RCTs), 8 cross-sectional studies, 6 survey-based studies, 1 interventional study, 1 cohort study, and 1 case-control study. Studies that used a qualitative design were used to assess the types of narratives (i.e., based on Arthur W. Frank’s illness narrative types – chaos-stories, restitution-stories, and quest-stories) shared by individuals with PD. Of these, 24 investigated ACP (Supplemental Table 1), while 18 focused on PC in PD (Supplemental Table 2).

### Knowledge of ACP or PC

Knowledge and awareness of ACP and PC among individuals with PD and their caregivers varied across studies. Specifically, insufficient information or incomplete understanding explained a reduced awareness or lower occurrence of ACP among n= 1929 out of 4154 individuals with PD (46.4%) in 10 out of 24 studies (McLaughlin et al. 2011; Boersma et al. 2016; Habermann and Shin 2017; Kluger et al. 2019; Lum et al. 2019; Churm et al. 2022a, 2022b; Seshadri et al. 2023; Lennaerts-Kats et al. 2024; Peabody et al. 2025). Individuals with PD and their caregivers often struggled to identify relevant questions regarding disease progression and future care, and individuals with PD frequently reported insufficient information about prognosis. Caregivers also noted a lack of information provided after diagnosis particularly regarding disease progression and future planning.

Regarding studies into PC, insufficient knowledge explained a lower occurrence of PC for *n* = 1564 individuals with PD in 13 out of 18 studies (Giles and Miyasaki 2009; Hasson et al. 2010; Fox et al. 2017; Klietz et al. 2018; Lennaerts-Kats et al. 2020, 2022; Prizer et al. 2020; Chen et al. 2023; Kundrick et al. 2023; Bock et al. 2024; Seshadri et al. 2024a, 2024b; Karmur et al. 2025). This lack of knowledge of ACP and PC can occur because medical decisions are made without consulting family caregivers or clinicians (Lum et al. 2019). Other factors contributing to this limited knowledge included unfamiliarity with ACP terminology, how to utilize advance directives (ADs; Churm et al. 2022b), inadequate understanding of disease prognosis (Clarke et al. 2018), and insufficient information about disease progression (Kurpershoek et al. 2021). Interestingly, a very small subset of individuals with PD (n= 30) who perceived a lack of interest from neurologists in advance planning were uncertain about completing an AD but also about the ACP process (Boersma et al. 2016). Regarding PC itself, some individuals with PD (n= 47) received minimal guidance or emotional support from their neurologists to manage their care (Prizer et al. 2020). In another small subset study of n= 10, individuals with PD misunderstood PC as “home help” or “counseling,” or mistakenly believed it was only for those with a cancer diagnosis (Fox et al. 2017). Some patients expressed a need for additional information about PC, particularly regarding ACP for end-of-life care (Klietz et al. 2018). Caregivers also noted a deficiency in sufficient information provided following diagnosis (McLaughlin et al. 2011), along with delays in recognizing the palliative stage of PD (Fox et al. 2016). In a study involving patients with late-stage PD (n= 10), participants prioritized present care over advance planning thereby expressing a desire to prolong their stay at home (Read et al. 2019).

### Timing of ACP or PC

The timing of ACP is often delayed and uncertain. Misconceptions that ACP or PC is only relevant when caregivers can no longer manage, led to late discussions thereby often delaying ACP until a crisis. In 9 out of 24 studies involving n= 1576 individuals with PD and n= 721 care partners or health care workers, the start of ACP discussions was delayed until the end of the disease trajectory (McLaughlin et al. 2011; Boersma et al. 2016; Fox et al. 2016; Lum et al. 2019; Read et al. 2019; Churm et al. 2022a, 2022b; Seshadri et al. 2023; Peabody et al. 2025). ACP was frequently neglected unless initiated by patients and some patients and caregivers viewed it mainly as completing ADs rather than discussing values and future care. Moreover, in 1 study involving a sample of 1266 individuals with PD, ACP was often disregarded unless initiated by the patient themselves (Seshadri et al. 2023). Even when individuals with PD do initiate the process, ACP discussions might still be overlooked by neurologists due to feelings of uncertainty (Boersma et al. 2016) or hesitation in individuals with PD (Read et al. 2019). Individuals with PD find it challenging to determine the right timing for ACP, viewing it as a future event that is linked to health changes (Churm et al. 2022a). This could explain why in 3 separate studies involving 240 individuals diagnosed with PD, there was a preference for initiating ACP early on (Tuck et al. 2015; Kluger et al. 2019; Kurpershoek et al. 2021). In the largest study of those 3 studies involving 106 individuals with PD, 37.7% believed that neurologists should initiate ACP discussions early in their illness (Kundrick et al. 2023).

In relation to PC, individuals with PD expressed a clear need for early information about disease progression. Many participants wanted future-oriented discussions with neurologists although opinions differed on the exact timing. Specifically 8 out of 18 studies involving n= 236 individuals with PD and n= 145 care partners or health care workers underscore such a delay in timing (Hasson et al. 2010; Fox et al. 2017; Lennaerts-Kats et al. 2020, 2022, 2024; Prizer et al. 2020; Kundrick et al. 2023; Seshadri et al. 2024b). Patients perceived that care planning relied on ad hoc rather than proactive approaches, leaving caregivers without a clear understanding of what to expect in the future. However, timely discussions in PC are preferred in 5 separate studies involving 178 individuals with PD and 10 caregivers (Lennaerts-Kats et al. 2020; Kundrick et al. 2023; Bock et al. 2024; Seshadri et al. 2024b; Karmur et al. 2025). This could be facilitated via more advance guidance (Seshadri et al. 2024b) or by providing more information about the disease’s progression (Bock et al. 2024). In a smaller study comprising 19 individuals with PD and 12 caregivers, those individuals with PD in early stages of disease tended to avoid thinking about the future, while those in advanced stages facing declining medication effectiveness, were more willing to consider ACP (Fox et al. 2017). Moreover, caregivers’ perceptions on PC are often hindered by misconceptions, such as viewing it as necessary only when they couldn’t cope (McLaughlin et al. 2011). As a result, discussions are often solely about how to construct ADs, limiting discussions on values (Lum et al. 2019). Moreover, the uncertainty in caregivers about the timing of ACP delays initiation of end-of-life discussions until calamities, despite recognizing the need for early planning (Fox et al. 2016).

### Perception of autonomy support in ACP or PC

When viewing autonomy as the capacity for self-determination, 11.4% of the individuals with PD in this systematic review (n= 472 out of a total of n= 4154) express a preference for initiating ACP or PC discussions themselves (Hudson et al. 2006; Kwak et al. 2014; Tuck et al. 2015; Fox et al. 2017; Clarke et al. 2018; Klietz et al. 2019; Churm et al. 2022b). This was particularly seen in a survey study involving 82 individuals with PD, for whom a significant motivation for creating ADs was to assert autonomy and responsibility for end-of-life care (Klietz et al. 2019). In contrast, most individuals with PD (n= 2258 out of a total of n= 4154 in 23 out of 42 studies) suggest they require more autonomy support to start discussions about ACP or PC (Hasson et al. 2010; McLaughlin et al. 2011; Boersma et al. 2016, 2017; Fox et al. 2016; Habermann and Shin 2017; Klietz et al. 2018; Lum et al. 2019; Read et al. 2019; Jordan et al. 2020; Kluger et al. 2020; Lennaerts-Kats et al. 2020, 2022, 2024; Prizer et al. 2020; Kurpershoek et al. 2021; Nicholas et al. 2021; Churm et al. 2022a; Chen et al. 2023; Kundrick et al. 2023; Seshadri et al. 2023, 2024b; Bock et al. 2024). Physicians can facilitate patient autonomy through actions such as providing disease progression details and participating in decision-making to help patients achieve their health objectives and increase their control over managing their condition (Arora et al. 2009). When ACP is conducted by general practitioners (GPs) or in collaboration with GPs and the patient’s family, PD care showed a significant reduction in hospital deaths (Nicholas et al. 2021). However, a small subset of individuals with PD (n= 145 out of 4154) often face inadequate support for autonomy, potentially due to neurologists’ lack of interest in ACP (1 study involving n= 30 individuals with PD) (Boersma et al. 2016), their primary focus on motor symptoms (Bock et al. 2024) and conflicting advice regarding the importance of ACP (Lum et al. 2019; Lennaerts-Kats et al. 2020, 2022; Seshadri et al. 2024b). Furthermore, 50 individuals with PD experience delays in neurologist appointments and their insufficient coordination among healthcare providers increases the emotional, social and financial burden of disease (McLaughlin et al. 2011; Prizer et al. 2020). This was also observed in studies concerning PC where a smaller study revealed that individuals with PD specifically expressed a need for attentive listening from their physician and a wish for more extensive discussions on end-of-life issues (Lennaerts-Kats et al. 2022). In a Chinese study, caregivers consistently stressed the significance of healthcare professionals acknowledging the dependency of individuals in advanced stages of PD on PC (Lennaerts-Kats et al. 2020). When neurologists appeared to overlook this aspect, general practitioners frequently stepped in to offer assistance through home visits (Hasson et al. 2010) or via hospital-based, multidisciplinary clinics (Giles and Miyasaki 2009).

### Caregivers’ involvement in surrogate decision-making concerning ACP or PC

In 6 out of 37 studies involving n= 674 individuals with PD, caregivers enhanced care (Tuck et al. 2015) and care planning (including increased or earlier ACP or the development of ADs with family cosignatures) (Churm et al. 2022a, 2022b; Fox et al. 2015; Klietz et al. 2019; Nicholas et al. 2021). In 2 studies, caregivers and individuals with PD (n= 40) collaborated in making difficult decisions (Read et al. 2019; Jordan et al. 2020). In one of those studies in which several individuals with PD completed an AD, they excluded their physician from the ACP process indicating a preference for management exclusively by the family (Boersma et al. 2016). Several caregivers (n= 256 out of 2191) also frequently feel responsible for making medical decisions (Kwak et al. 2014; Churm et al. 2022a) coordinating timely discussions about prognosis and future symptoms (Lennaerts-Kats et al. 2022) or assuming the role of care coordinator (Bock et al. 2024). However, they often encounter challenges in discussing advanced disease stages with neurologists (Habermann and Shin 2017), often due to emotional distress (Seshadri et al. 2024a), limited awareness of the patient’s preferences (Fox et al. 2017; Lennaerts-Kats et al. 2020) or the lack of knowledge of PD course and the possibility of ACP (Habermann and Shin 2017) or PC (McLaughlin et al. 2011). With regard to care planning, barriers within relationships can hinder ACP (Lum et al. 2019), while the presence of family is reason enough to disregard ACP (Churm et al. 2022b). Still, in a significant study involving 267 individuals with PD, most (56.6%) indicated a preference for early involvement of their family in the management of the progression of illness (Tuck et al. 2015). Another study involving 82 individuals with PD demonstrated that such early involvement could result in ADs with either a spouse or a family physician co-signing (Klietz et al. 2019).

### Types of narratives shared by individuals with PD in studies on ACP or PC

Arthur W. Frank identifies 3 types of illness narratives (Frank 2013). Restitution stories show illness as temporary, focusing on getting better and returning to normal life. Chaos stories describe the confusion and disorder that illness brings, showing how out of control it can feel. Quest stories are accepting illness and trying to find meaning or personal growth through the experience. Most of the identified narrative types narrated by individuals with PD and caregivers were chaos narratives. They appeared in 8 studies on ACP (McLaughlin et al. 2011; Boersma et al. 2016, 2017; Klietz et al. 2019; Kluger et al. 2019; Jordan et al. 2020; Churm et al. 2022b; Seshadri et al. 2023) involving n= 1572 individuals with PD and 9 studies on PC involving n= 140 individuals with PD (Hudson et al. 2006; Giles and Miyasaki 2009; Hasson et al. 2010; Fox et al. 2017; Badger et al. 2018; Klietz et al. 2018; Lennaerts-Kats et al. 2020; Seshadri et al. 2024a, 2024b). Quest narratives were found in 5 studies on ACP involving n= 330 individuals with PD (Tuck et al. 2015; Clarke et al. 2018; Lum et al. 2019; Read et al. 2019; Kurpershoek et al. 2021) and 4 studies into PC involving n= 83 individuals with PD (Prizer et al. 2020; Lennaerts-Kats et al. 2022; Chen et al. 2023; Bock et al. 2024). Quest narratives were identified in qualitative studies among individuals with PD who aimed to initiate ACP at the earliest opportunity (Tuck et al. 2015; Kurpershoek et al. 2021) to uphold their identity despite PD (Read et al. 2019), or sought to independently navigate resources to address nonphysical needs (Prizer et al. 2020). In studies assessing PC, quest narratives became apparent when it was observed that individuals with PD had limited comprehension of PC but showed an interest in it (Chen et al. 2023), felt obliged to communicate healthcare information to their providers (Prizer et al. 2020) or sought honest discussions with healthcare professionals about their health (Lennaerts-Kats et al. 2022).

### Effectiveness of intervention studies to improve ACP or PC

Interventions evaluating counseling to support the ACP process show that patients and caregivers report positive experiences leading to a better understanding of the current health situation, future care needs and end-of-life decisions. Specifically in the study by Lennaerts-Kats et al., patients and caregivers describe the counseling as supportive and helpful in gaining a clearer understanding of their current health status and future care needs including end-of-life decisions (Lennaerts-Kats et al. 2024). Kluger and co-authors have investigated whether an outpatient PC intervention enhances outcomes for individuals with PD and caregivers compared to standard care (Kluger et al. 2020). In the PC intervention group, this led to better quality of life (QOL) and individuals with PD were more likely to have completed ADs (67.0%, n= 59 of 88 versus 30.3%, n= 23 of 76 in the control group) (Kluger et al. 2020). Other significant improvements following the PC intervention were a reduced motor symptom severity, lower non-motor symptom burden, decreased caregiver anxiety and alleviated caregiver burden at 12-months (Kluger et al. 2020). In a secondary analysis of the same trial, the authors used longitudinal regression models to study changes in QOL of individuals with PD and care partners, while Spearman correlations were used to evaluate the correlates of score changes in outcomes (Kluger et al. 2020). This identified that individuals with PD reported (among others) improvements in anxiety, well-being, stiffness and total symptom burden, all of which correlated significantly with enhanced QOL (Bock et al. 2022). Moreover, reductions in patient anxiety, depression, and grief throughout the study period were also significantly associated with improvements in patient QOL (Bock et al. 2022).

## Discussion

This systematic review with narrative synthesis identified that limited knowledge or incomplete understanding of ACP and PC explains its lower occurrence among individuals with PD (n= 1929 out of 4154, 46.4%). Therefore, one could argue that most individuals with PD expressed a need for greater autonomy support to initiate ACP or PC discussions (n= 2258 out of 4154 individuals with PD across 23 of 42 studies). Furthermore, the timing of ACP is often delayed and uncertain due to misconceptions that ACP or PC is only relevant when caregivers can no longer manage. Among 1565 individuals with PD (37.6%) and 721 care partners or health care workers (33%), the start of ACP discussions was delayed until the end of the disease trajectory. Lastly, the assessment of narrative types shared by individuals with PD in qualitative studies predominantly emphasizes chaos stories that underscore inadequate autonomy support. Caregivers however frequently prioritize early participation in medical decisions which could potentially result in the implementation of ADs being co-signed by a spouse or family physician or completed without the treating physician’s involvement. Although several individuals with PD express a preference for initiating ACP or PC discussions themselves, more studies are needed to investigate whether patient autonomy in PD is improved by participation of caregivers or neurologists in ACP or PC.

The principle of autonomy in biomedical ethics includes a “notion of the self, which is to be respected, left unmanipulated and which is, in certain ways, independent and self-determining” (Dworkin 2015). According to Beauchamp and Childress, autonomous actions or decisions are characterized by intentionality, non-control and understanding (Beauchamp and Childress 2001). Patients’ decisions are autonomous when deliberate, free from influences diminishing self-direction and fully understood (Beauchamp and Childress 2001). The deficiency in understanding and delay in timing of ACP or PC however is potentially reducing autonomous decision making for individuals with PD. Often the delay in timing can be attributed to the fact that neurologists prioritize motor symptoms, potentially neglecting ACP and offering conflicting advice on its importance. In contrast, incorporating early ACP (which could lead to the development ADs) was previously found to enhance alignment between preferences for care and delivered care and increased the frequency of end-of-life care discussions between patients and healthcare professionals (Houben et al. 2014). Importantly, neurologist-led counseling increases the accuracy of ADs by increasing awareness of specific complications and treatment options throughout the progression of the disease (Klietz et al. 2019). Another study revealed that ACP was both more prevalent and more effective in reducing hospital deaths (Nicholas et al. 2021). Given that a valid AD holds the same authority as decisions made by competent individuals, the decision to create one for future decisions should be made by a competent individual. Therefore, it can be argued that these decisions should be made early in the disease trajectory of PD and require (autonomy) support from neurologists or caregivers. This is crucial for 2 reasons. First, the premise that a competent individual is the most reliable evaluator of own interests weakens when it comes to decisions concerning future care (Buchanan and Brock 1989). Second, therapeutic options and a person’s prognosis can change between the issuance of the AD and its intended implementation (Buchanan and Brock 1989). This underscores the importance of creating detailed and specific ADs to specify the preferences of individuals with PD for critical medical decisions.

**Figure 2.**
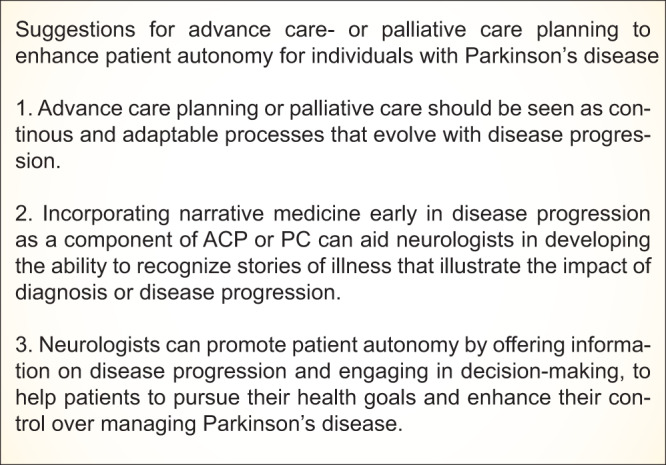
Suggestions in care planning for individuals with Parkinson’s disease.

The relevance and implication of this study is 3-fold (Figure 2). First, ACP or PC planning shouldn’t be delayed until the final stages of PD. Instead of treating it as an outcome, ACP or PC should be viewed as ongoing processes that adapt to disease progression. When neurological care and QoL for individuals with PD diverge, it should lead to interventions that were shown to alleviate motor symptom severity and non-motor symptom burden (Kluger et al. 2020). Second, integrating narrative medicine early in the disease progression as part of ACP or PC preparation can assist neurologists in developing the ability to recognize stories of illness (Charon 2008) and could facilitate readiness to engage in ACP for patients (Jiao et al. 2024). However, more studies are needed to investigate this. Third, a suggestion could be that neurologists can promote patient autonomy by offering information on disease progression and engaging in decision-making, thereby helping patients to pursue their health goals and enhance their control over managing their condition (Figure 2). In a previous systematic review of ACP in PD, it was argued that ACP discussions “should be individualized in content, timing and approach” (Nimmons et al. 2020). Our narrative synthesis of studies indicates that early recognition of narratives from individuals with PD highlights stories emphasizing the loss of self-perceived autonomy. Often this is due to a decreased understanding of PD progression and delayed initiation of ACP or PC planning. However, narrative medicine programs in clinical practice have previously been shown to enhance skills such as relationship-building, empathy and confidence (Remein et al. 2020). Therefore, their implementation in PD care, which could lead to PC interventions, can aid in recognizing the importance of early communication with families regarding PD’s incurable nature, thereby creating realistic expectations for managing disease progression.

This study has some limitations. Although we included 42 studies that investigated ACP or PC planning in PD, most qualitative studies are thematic analyses with small sample sizes. Moreover, many studies concentrated on individuals with PD from Western, industrialized nations like the US, the Netherlands, Ireland, and Germany, possibly restricting the generalizability of the results. Nonetheless, several suggestions can be proposed for future research, which should focus on (i) the impact of providing more adequate information to individuals with PD on the benefit of ACP or PC planning; (ii) whether the lack of understanding in individuals with PD and delay in ACP or PC timing diminish autonomous decision-making among individuals with PD; (iii) whether support for self-perceived autonomy primarily comes from the treating neurologist or caregiver; and (iv) whether the integration of narrative medicine could inform advance or PC planning and interventions in PD (Trahair and Mantri 2023).

We conclude that delays or lack of awareness in ACP and PC discussions can compromise self-perceived autonomy. Integrating narrative medicine into PD care could help neurologists initiate advance care and palliative planning earlier.

## Supporting information

10.1017/S1478951526103277.sm001 Bouma et al. supplementary material Bouma et al. supplementary material

## Supplementary material

The supplementary material for this article can be found at https://doi.org/10.1017/S1478951526103277.

## Figure 1 Long description

The flowchart outlines a systematic literature search conducted on February 2nd, 2026, starting with 377 published papers sourced from PubMed (114), Embase (85), Web of Science (80), Cochrane Library (19), CINAHL (49) and PsycINFO (30). The process includes the exclusion of 121 studies for not meeting criteria. Additionally, 214 full-text articles are excluded for various reasons: 136 reviews, 7 case reports, 30 protocol/opinion/guideline articles, 8 atypical Parkinsonian studies, 5 COVID-19 related studies, 12 non-English articles, 9 articles on neurologists′ views, 4 unavailable as full text and 3 trial descriptions. Finally, 42 full-text articles are assessed for eligibility and included in qualitative synthesis.

Navigate back to Figure 1.
