# Supplementary material for: Patient autonomy in advance care planning for Parkinson’s disease: Systematic review with narrative synthesis
Source: Palliat Support Care. 2026 Jul 23;24:e204. doi: 10.1017/S1478951526103277 (PMC13430485; doi:10.1017/S1478951526103277)
Supplement: Bouma et al. supplementary material [file S1478951526103277sup001.zip › S1478951526103277sup001/Revised Tabel 1.docx]

| **Supplemental Table 1.** Characteristics of studies evaluating advanced care planning in Parkinson’s disease | | | | | | | | | | |  |
| --- | --- | --- | --- | --- | --- | --- | --- | --- | --- | --- | --- |
|  | **Study**  **design** | **Cases**  **(n)** | **Caregivers,**  **or controls (n)** | **Study**  **Objective** | **Knowledge or frequency of ACP** | **Timing of ACP** | **Surrogate decision making** | **Autonomy support** | **Narrative**  **type** | **Quality appraisal**  **(CASP)** |  |
| McLaughlin et al. Palliat Med. 2011 Mar;25(2):177-182. (1) | Exploratory, qualitative study |  | 26 caregivers | To explore the experience of informal carers of people with PD. | Carers noted the insufficient information provided after diagnosis. They expressed specific information needs, including understanding the progression to prepare for the future. | Discussions were hindered by misconceptions about palliative care, particularly the belief that it was only relevant when caregivers couldn't manage anymore. Patients had no contact with palliative care services according to caregivers. | Carers struggled to discuss the advanced stage of the disease and end-of-life matters with healthcare professionals due to a lack of awareness about palliative care services and misconceptions about hospice care, often deeming palliative care unnecessary at this stage. Bovenkant formulier | Although GPs were valued, some felt their knowledge of PD was limited. Consequently, carers leaned towards neurologists for ongoing care. However, delays in specialist reviews and a lack of coordination among service providers had negative effects on both patients and carers. | Chaos story | 9/10 |  |
| Lum et al. Neurology 2019 May 28;92(22): e2571-e2579. (2) | Qualitative descriptive  study within a RCT of neuro-palliative care compared to standard care | 30 | 30  care-partners | To explore patient and care partner perspectives on ACP to shape a patient/care partner-centered clinical framework. | Patients faced a barrier to ACP due to a lack of complete understanding, sometimes making medical decisions without understanding choices or discussing them with decision-makers or clinicians. | Some patients and care partners saw ACP as merely completing advance directives, limiting discussions on life values and the document's implications. | At the patient and care partner level, relational barriers hindered discussions about future medical care planning, as both encountered resistance from the other party. | Some noted insufficient physician support, conflicting messages on ACP importance, and time constraints during clinic visits. | Quest  story | 10/10 |  |
| Clarke et al. BMC Neurol. 2018 Aug 16;18(1):115. (3) | Longitudinal qualitative interview study | 3 | 3 | Study how individuals with PD and family members decide on future care as the condition worsens. | Individuals with PD couldn’t articulate their prognosis which hindered the initiative to prepare for the future. | No correlation between adopting an ACP strategy and the need for information. | Those with PD and caregivers sought varied information and support options to match individual preferences and disease stages, rejecting a one-size-fits-all approach. | Some wanted more involvement in care planning and felt frustrated by the scarcity of available information, | Quest  story | 9/10 |  |
| Fox et al. BMC Palliat Care. 2016 Feb 9:15:15. (4) | Qualitative, exploratory inductive study |  | 30 caregivers | To examine caregivers diverse views on palliative care for Parkinson's patients, | Caregivers noted a delay in identifying the palliative stage of PD compared to other neurological illnesses, citing difficulty in defining end-of-life phases in PD. | Uncertainty about when to introduce palliative care often delayed its initiation until a crisis, despite the recognized need for early planning. | HCWs stressed the need to inform families early on about PD’s incurability, setting realistic expectations to cope with disease progression. | HCW outside of palliative care may feel "uncomfortable" or "afraid" discussing palliative care, with some actively avoiding such conversations with patients and families. | NA | 10/10 |  |
| Kwak et al. South Med J. 2014 Mar;107(3):178-85. (5) | Cross-sectional survey study. | 60 | 64 spouses and adult children | Investigate advance care planning and proxy decision-making for advanced PD patients by family healthcare proxies. |  | NA | Proxies favored shared decision-making with other family members, but 42% were unaware of patients' preferences for life support treatments. | Only 38% shared the AD with physician.  Sharing advance directives with a physician led to a preference for hospice care. | NA | 10/10 |  |
| Kurpershoek et al. Front. Neurol. 2021 (6) | Qualitative study design and data-driven approach. | 20 | NA | Study experiences, needs, and preferences regarding the content and timing of ACP. | Individuals with PD often felt uninformed about the progression of disease. | ACP shortly after the diagnosis |  | Healthcare provider should initiate ACP conversation.  Neurologist focused mainly on medication and didn't have time for a holistic approach to living disease. | Quest story | 8/10 |  |
| Tuck et al. Am J Hosp Palliat Care. 2015 Feb;32(1):68-77. (7) | Cross-sectional survey study. | 267 | NA | PHQ-2 and 7-item binary ‘‘Information’’ subscale of the Krantz Health Opinion Survey | Most individuals with PD (183 out of 267, 68.5%) had ACP documents; completion rates were higher for those recently diagnosed (less than 5 years, 42 out of 47, 89.3%). | Early in the course of the disease (50%) | Majority prefers involving family early in the disease. (56.6%) | Those desiring more control preferred to initiate the ACP discussion.  Patients (74.5%) should initiate discussion on ACP. | Quest story | 10/10 |  |
| Kluger et al. *JAMA Neurol.*2020;77 (5):551-560. (8) | Randomized controlled trial | 210 | 175 caregivers | Study if outpatient palliative care improves outcomes for PD individuals and caregivers compared to standard care. | NA | NA | Individuals in the PC intervention group were more likely to have completed state-specific advance directives 67.0% (59 of 88), compared to 30.3% (23 of 76) | Individuals in the PC intervention group were more likely to have filed paperwork with their practitioners. | NA | 9/10 |  |
| Klietz et al. Parkinsons Dis. 2019 Aug 4:2019:2107821. (9) | Qualitative and quantitative cross-sectional survey study. | 82 | NA | Questionnaire about  their AD | NA | ADs were generally specific but lacked specificity for PD. | 21.9% of ADs had a spouse cosign, and 14.6% had a family physician cosign. | Reason for AD: it expressed autonomy and responsibility for end-of-life care | Chaos story | 10/10 |  |
| Kluger et al. JAMA Neurol. 2024 Jan 1;81(1):39-49. (10) | Non-blinded, comparative effectiveness trial. | 300 | 300 | Evaluate if PC training and remote PC team access improve outcomes for PDRD patients in community settings. | NA | PC training enhanced the completion of advance directives. | NA | NA | NA | 10/11 |  |
| Seshadri et al. Neurol Clin Pract. 2023 Dec;13(6): e200214. (11) | Cross-sectional study using electronic surveys. | 1266 | 616 | Study how individuals with PD and caregivers view their palliative care satisfaction clinical communication quality. | 85% of individuals with PD, indicated a level of knowledge about palliative care (PC), while 68% expressed that the primary objective of PC was to assist their friends and family in coping with the illness. | ACP was neglected (79%) unless the patient initiated it. | Care partners' well-being (57%) was only addressed if raised by the patient. | 30% of individuals with PD received help from doctors with disease uncertainties.  64% of individuals with PD found clinical communication to be open and honest. | Chaos story | 9/10 |  |
| Churm et al. Eur Geriatr Med. 2022 Feb;13(1):109-117. (12) | Qualitative study with semi-structured interviews | 33 | 19 | Explore Parkinson's patients and their relatives' views on future and care planning. | Interviewees had limited ACP knowledge, grappling with terminology and AD understanding. | Interviewees saw ACP as new. Their views could change with time and increased awareness. | Family considerations were crucial in decision-making. While some made choices based on familial obligations, others found the presence of family reasons enough to disregard ACP. | Few interviewees shared preferences with health professionals, necessitating a more proactive role for healthcare providers in initiating ACP discussions. | Chaos story | 10/10 |  |
| Churm et al. J Geriatr Psychiatry Neurol. 2022 Jan;35(1):168-175. (13) | Mixed methods study with a postal quantitative survey and qualitative semi-structured interviews | 196 | NA | Explore the views of people with PD and their relatives on ACP and future plans. | Most respondents hadn't heard of ACP. Many wanted more info, preferably at home from someone they knew. Only 13% had made or considered future care wishes; 70% hadn't discussed this with anyone. | Individuals with PD had difficulty determining the right time for ACP, seeing it as a future event, possibly tied to changes in health or understanding their condition better. | Family members consistently expressed a sense of responsibility for making medical decisions. | Both individuals with PD and their relatives were highly engaged with PD nurse specialists due to their approachability, openness, empathy, and responsiveness. In contrast, there were concerns about the accessibility of medical doctors within the team and the frequency of contact with them. | NA | 9/10 |  |
| Nicholas et al. BMJ Support Palliat Care. 2021 Sep 6:bmjspcare-2021-003105. (14) | Retrospective, observational, comparative cohort study | 96 | 95 | Study factors affecting the place of death in individuals with Parkinson's disease (PD). | Individuals with PD had a higher ACP frequency than controls. | No significant difference in timing of APC when compared to controls or MS patients. | More ACPs (65.7%, 44 out of 67) was implemented by GPs and/or the patient's family in individuals with PD compared to controls. | ACPs implemented by GPs or with the collaboration of GPs and the patient's family led to significantly fewer hospital deaths. | NA | 10/10 |  |
| Jordan et al. Ann Palliat Med. 2020 Feb;9(Suppl 1):S63-S74. (15) | Qualitative descriptive secondary analysis  from a clinical trial. | 30 | 30 | Explore PD patient and care partner needs related to future planning using a palliative care framework | Both patients and care partners struggled with identifying important questions and information related to disease. | NA | Care partners sought comprehensive support beyond physical changes in PD patients, considering factors like housing, driving, function, and finances. | Both individuals with PD and their caregivers relied on peers in support groups as guides for anticipating future developments. | Chaos story | 10/10 |  |
| Read et al. PLoS One. 2019 Dec 30;14(12):e0226916. (16) | Semi-structured open-ended questions, and qualitative thematic analysis. | 10 | NA | Explore the healthcare experiences and unmet care needs of individuals with late-stage PD | Participants prioritized current care over ACP expressing a desire to remain in their homes as long as possible. | There was a hesitation and ambivalence to address discussions about the future, end-of-life, or palliative care. | Those with advanced PD seldom initiated care. Family members organized doctor's appointments and served as mediators with healthcare professionals during crises. | Individuals with PD faced a lack of coordination and continuity in treatment and care, especially during hospital admissions. | Quest story | 10/10 |  |
| Kluger et al. Mov Disord Clin Pract. 2018 Nov 16;6(2):125-131. (17) | Cross‐sectional observational study | 90 | 47 patients with advanced cancer | Assess the prevalence of key symptomatic, psychosocial, and spiritual issues in PD and their influence on HRQOL. | 50 individuals (56%) completed an AD after hearing about ACP, while 21 (23%) did not, and 14 (16%) were unaware of ACP. | In 72 individuals with PD (80%), the preferred timing for ACP was before illness onset, while 11 (12%) preferred discussing it at diagnosis. | More individuals with PD completed medical power of attorney paperwork compared to persons with advanced cancer (76% vs. 53%; P = 0.018). | In 52 individuals with PD (58%), the primary care physician was preferred to bring along, while 49 (54%) chose their neurologist. | Chaos story  (Spiritual well-being impacted HRQOL). | 7/10 |  |
| Gillard et al. Parkinsonism Relat Disord. 2019 May:62:73-78. (18) | Cross-sectional prospective study | 50 PD | 50 caregivers  50 healthy controls | To determine if parkinsonian disorders affect the likelihood of having advance directives (four were compared) | 53% of controls, 68% of PD group, 86% of APD group, and 76% of caregivers had at least one AD. | NA | Individuals with PD were four times more likely to have a durable power of attorney and twice as likely for healthcare. APD participants showed similar trends. | NA | NA | 10/10 |  |
| Boersma et al. Neurol Clin Pract. 2016 Jun; 6(3): 209–219. (19) | Cross sectional qualitative study | 30 | 10 caregivers | To understand PD patients' unmet palliative care needs and their preferences. | Individuals with PD were unsure about having an advance directive and expressed uncertainty about the process. | Individuals with PD expressed worries about ACP and advanced directives, often feeling unsure about where to seek guidance. | Several individuals with PD, having completed an AD, excluded their physician from the process, suggesting that it should be managed by either their family or a lawyer. | Individuals with PD felt neurologists lacked interest in advance care planning. | Chaos story | 9/10 |  |
| Boersma et al. J Palliat Med. 2017 Sep;20(9):930-938. (20) | Qualitative semi-structured interviews. | 11 | 11 caregivers | To understand the needs and care preferences of PD caregivers within a palliative care framework. | Many expressed concerns about future issues not addressed in ADs. | NA | Caregivers favored personalized care models in team-based clinics but had reservations about adding more clinicians. | Most caregivers had ADs but desired more involvement and guidance from their healthcare teams. | Chaos story | 10/10 |  |
| Dijkstra et al. Mov Disord Clin Pract. 2025 Dec 3. (21) | Questionnaire and electronic health records. | 182 | NA | To explore existing documentation practices for end-of-life (EoL) care across Europe to inform the development of evidence-based guidelines. | EoL care preferences were documented for 35 of 173 participants (20%), with only 12 (7%) recorded in hospital or general practice EHRs. | NA | NA | The most documented preferences were appointing a legal representative. | NA | 9/10 |  |
| Habermann et al. J Clin Nurs. 2017 Jun;26(11-12):1650-1656. (22) | | Qualitative study | 14 | 14 spouses | To explore how couples with Parkinson’s disease discuss  their needs, concerns and preferences at the advanced stages of illness. | Significant lack of information for future planning. People with PD and their spouses often asked doctors what to expect but were usually told the disease progresses differently for everyone. While patients generally accepted this, spouses found it frustrating because it made planning difficult. | Spouses felt unprepared to discuss or decide on future options and their timing because they were not receiving enough conversations or clear information about prognosis from healthcare providers. | Because they were not given clear information about prognosis, many couples did not make plans or decisions for the future. Over half had no ADs, wills, or health care power of attorney in place. | Although some asked doctors about disease progression, they were usually told it was unpredictable. Patients often accepted this but spouses found it frustrating. | NA | 8/10 |
| Lennaerts-Kats et al. J Neurosci Nurs. 2024 Oct 1;56(5):174-179. (23) | | Interventional prospective, pretest/posttest feasibility  study.  The intervention consisted of 3 to 6 counseling sessions with the patient and family caregiver  with the main aim to support the ACP process and coordinate  care. | 20 | 11 family caregivers | To assess the feasibility and acceptability of the study processes to inform a larger randomized  controlled trial, aiming the effectiveness of a combined intervention on ACP and care coordination for people  with PD. | In total, 8 new types  of ACP documentation were added after intervention, which consisted  mainly of “no cardiopulmonary resuscitation” (41.7%),  “power of attorney” (20.8%), and “advanced directive”  (16.7%). | For some patients, it was the first time they had considered future care, while others had already discussed these issues with family members or their general practitioner. | NA | Patients and caregivers reported a positive experience with the intervention, describing it as both supportive and confronting. | NA | 8/10 |
| Peabody et al. Clin Park Relat Disord. 2025 Aug 9:13:100388. (24) | | Crossectional survey study | 250 |  | To investigate disparities in access to ACP based on race and/or ethnicity among a largely Hispanic PD population. | Hispanics were more likely to report not having heard of AD (61.7% vs 38.1%), while similar proportions in both groups reported hearing about but not completing AD (29.6% vs 23%). | Compared to Hispanics, more non-Hispanics preferred discussing ACP before getting sick while healthy (57.5% vs 39.1%). More Hispanics preferred discussing it when dying (8.7% vs 3.9% p = 0.210). | Both groups most often preferred a neurologist to initiate ACP discussions (69.0% Hispanics; 68.5% non-Hispanics), followed by primary care physicians (48.3% vs 63.8%), psychologists (24.1% vs 18.9%), patient advocates (14.7% vs 24.4%), and social workers (16.4% vs 15.0%). | NA | NA | 9/10 |
| \| PD, parkinson’s disease; EOL, end of life care; ACP, advance care planning; RCT, randomized controlled trial; ADs, advance directive \| \| --- \| | | | | | | | | | | | |

**References**

1. McLaughlin D, Hasson F, Kernohan WG, Waldron M, McLaughlin M, Cochrane B, et al. Living and coping with Parkinson's disease: perceptions of informal carers. Palliat Med. 2011;25(2):177-82.

2. Lum HD, Jordan SR, Brungardt A, Ayele R, Katz M, Miyasaki JM, et al. Framing advance care planning in Parkinson disease: Patient and care partner perspectives. Neurology. 2019;92(22):e2571-e9.

3. Clarke G, Fistein E, Holland A, Tobin J, Barclay S, Barclay S. Planning for an uncertain future in progressive neurological disease: a qualitative study of patient and family decision-making with a focus on eating and drinking. BMC Neurol. 2018;18(1):115.

4. Fox S, Cashell A, Kernohan WG, Lynch M, McGlade C, O'Brien T, et al. Interviews with Irish healthcare workers from different disciplines about palliative care for people with Parkinson's disease: a definite role but uncertainty around terminology and timing. BMC Palliat Care. 2016;15:15.

5. Kwak J, Wallendal MS, Fritsch T, Leo G, Hyde T. Advance care planning and proxy decision making for patients with advanced Parkinson disease. South Med J. 2014;107(3):178-85.

6. Kurpershoek E, Hillen MA, Medendorp NM, de Bie RMA, de Visser M, Dijk JM. Advanced Care Planning in Parkinson's Disease: In-depth Interviews With Patients on Experiences and Needs. Front Neurol. 2021;12:683094.

7. Tuck KK, Brod L, Nutt J, Fromme EK. Preferences of patients with Parkinson's disease for communication about advanced care planning. Am J Hosp Palliat Care. 2015;32(1):68-77.

8. Kluger BM, Miyasaki J, Katz M, Galifianakis N, Hall K, Pantilat S, et al. Comparison of Integrated Outpatient Palliative Care With Standard Care in Patients With Parkinson Disease and Related Disorders: A Randomized Clinical Trial. JAMA Neurol. 2020;77(5):551-60.

9. Klietz M, Öcalan Ö, Schneider N, Dressler D, Stiel S, Wegner F. Advance Directives of German People with Parkinson's Disease Are Unspecific in regard to Typical Complications. Parkinsons Dis. 2019;2019:2107821.

10. Kluger BM, Katz M, Galifianakis NB, Pantilat SZ, Hauser JM, Khan R, et al. Patient and Family Outcomes of Community Neurologist Palliative Education and Telehealth Support in Parkinson Disease. JAMA Neurol. 2024;81(1):39-49.

11. Seshadri S, Dini M, Macchi Z, Auinger P, Norton SA, Holtrop JS, et al. Reach of Palliative Care for Parkinson Disease: Results From a Large National Survey of Patients and Care Partners. Neurol Clin Pract. 2023;13(6):e200214.

12. Churm D, Dickinson C, Robinson L, Paes P, Cronin T, Walker R. Understanding how people with Parkinson's disease and their relatives approach advance care planning. Eur Geriatr Med. 2022;13(1):109-17.

13. Churm D, Dickinson C, Robinson L, Paes P, Cronin T, Walker R. How Do Patients With Parkinson's Disease Approach Advance Care Planning? A UK-Based Mixed Methods Study. J Geriatr Psychiatry Neurol. 2022;35(1):168-75.

14. Nicholas R, Nicholas E, Hannides M, Gautam V, Friede T, Koffman J. Influence of individual, illness and environmental factors on place of death among people with neurodegenerative diseases: a retrospective, observational, comparative cohort study. BMJ Support Palliat Care. 2021.

15. Jordan SR, Kluger B, Ayele R, Brungardt A, Hall A, Jones J, et al. Optimizing future planning in Parkinson disease: suggestions for a comprehensive roadmap from patients and care partners. Ann Palliat Med. 2020;9(Suppl 1):S63-s74.

16. Read J, Cable S, Löfqvist C, Iwarsson S, Bartl G, Schrag A. Experiences of health services and unmet care needs of people with late-stage Parkinson's in England: A qualitative study. PLoS One. 2019;14(12):e0226916.

17. Kluger BM, Shattuck J, Berk J, Sebring K, Jones W, Brunetti F, et al. Defining Palliative Care Needs in Parkinson's Disease. Mov Disord Clin Pract. 2019;6(2):125-31.

18. Gillard DM, Proudfoot JA, Simões RM, Litvan I. End of life planning in parkinsonian diseases. Parkinsonism Relat Disord. 2019;62:73-8.

19. Boersma I, Jones J, Carter J, Bekelman D, Miyasaki J, Kutner J, et al. Parkinson disease patients' perspectives on palliative care needs: What are they telling us? Neurol Clin Pract. 2016;6(3):209-19.

20. Boersma I, Jones J, Coughlan C, Carter J, Bekelman D, Miyasaki J, et al. Palliative Care and Parkinson's Disease: Caregiver Perspectives. J Palliat Med. 2017;20(9):930-8.

21. Dijkstra BW, Muente C, Garon M, Rosqvist K, Scharfenort M, Lex KM, et al. Documented End-of-Life Preferences of People With Parkinson's Disease or Parkinsonism Across Europe. Mov Disord Clin Pract. 2025.

22. Habermann B, Shin JY. Preferences and concerns for care needs in advanced Parkinson's disease: a qualitative study of couples. Journal of Clinical Nursing (John Wiley & Sons, Inc). 2017;26(11-12):1650-6.

23. Lennaerts-Kats H, Daeter L, Forkink A, Hukema RK, Bloem BR, Vissers KCP, et al. Implementing Advance Care Planning and Care Coordination in the Care for People With Parkinson Disease: A Feasibility Study. J Neurosci Nurs. 2024;56(5):174-9.

24. Peabody T, Abou-Ezzi M, Hernandez L, Moore H, Vargas-Parra S, Cruz A, et al. Advance care planning in Hispanic populations with Parkinson's Disease: Investigating disparities in end-of life care. Clin Park Relat Disord. 2025;13:100388.

25. Jiao J, Brumbach BH, Lobb B, Goldhirsch S, Hiller A. Narrative Medicine Interventions for Advance Care Planning in Parkinson's Disease. J Palliat Med. 2024;27(8):984-5.
