# Supplementary material for: Patient autonomy in advance care planning for Parkinson’s disease: Systematic review with narrative synthesis
Source: Palliat Support Care. 2026 Jul 23;24:e204. doi: 10.1017/S1478951526103277 (PMC13430485; doi:10.1017/S1478951526103277)
Supplement: Bouma et al. supplementary material [file S1478951526103277sup001.zip › S1478951526103277sup001/Revised Tabel 2.docx]

| **Supplemental Table 2.** Characteristics of studies evaluating palliative are in Parkinson’s disease | | | | | | | | | | |
| --- | --- | --- | --- | --- | --- | --- | --- | --- | --- | --- |
|  | **Study**  **design** | **Cases**  **(n)** | **Carers or**  **controls**  **(n)** | **Study**  **Objective** | **Knowledge or frequency of PC** | **Timing of PC** | **Surrogate decision making** | **Autonomy support** | **Story type** | **Quality appraisal**  **(CASP)** |
| Bock et al. Palliat Med. 2024 Feb;38(2):240-250. (1) | Qualitative study in RCT | 28 | 33 | To outline the encounter with an innovative community-based palliative care approach for PD | NA | Multiple participants expressed the need for advance information about the progression of the disease. | Oftentimes there was a need for care partners to serve as care coordinator | Care by neurologists was too focused on motor symptoms, with primary neurologists being efficient yet sometimes lacking understanding of the full range of needs in PD | Quest story | 10/11 |
| Seshadri et al. Am J H Palliat Care. 2024 Jan 24. PMID: 38264847 (2)  . | Qualitative descriptive research design | NA | 16 | To understand family members' perception of their role, and of the challenges and rewards of PD caregiving. | NA | NA | Disease progression and various symptoms increase emotional distress for care partners. They feel unprepared and saddened by both the patient's and their own losses. | Caring was considered a natural part of the partnership, evolving from a longstanding friendship. As the disease progressed, caregivers prioritized preserving the dignity of the individual with PD. | Chaos story | 10/10 |
| Seshadri et al. Parkinsonism Relat Disord. 2024 Feb: 119:105982.   (3) | Qualitative descriptive research design, with semi-structured interviews | 47 | 47 | To comprehend patients' and care partners' perceptions of palliative care and their needs before its implementation. | Most individuals with PD misunderstood palliative care regarding it as end-of-life care and not necessary in early stages. Some associated it with comfort in incurable diseases like cancer, while others confused it with hospice. | Individuals with PD expressed a desire for anticipatory guidance and discussions about the future with their neurologists, despite differing opinions on the timing of ACP. | NA | Individuals with PD sought more guidance from neurologists on PD and had differing views on when to discuss future planning; some preferred delaying such conversations after diagnosis, while others preferred early engagement with follow-up discussions. | Chaos story | 9/10 |
| Chen et al. BMC Health Serv Res 2023 Mar 6;23(1):215. (4) | Qualitative semi-structured interviews | 8 | 5 | To identify the obstacles and enhancers impacting patient-centered services for individuals with PD | Individuals with PD and family members possessed limited understanding of PC, yet they expressed an interest in it. | NA | Despite traditional beliefs about life and death, most individuals with PD and carers were willing to consider PC. | Several patients and caregivers highlighted the necessity for social support, a need that can be met through the palliative care program. | Quest story | 8/10 |
| Lennaerts-Kats et al. J Parkinsons Dis. 2022; 12(1): 207–219. (5) | Mixed methods case study design (qualitative and survey study) | 10 | 8 | To explore needs of patients with PD in the palliative phase and of their family caregivers. | Some individuals with PD hadn't thought much about advance directives or end-of-life decisions. | NA | Individuals with PD and family caregivers wanted timely discussions on prognosis, future symptoms, treatment, and reimbursement.  Bovenkant formulier  Onderkant formulier | Individuals with PD wanted more time for discussions with neurologists, especially regarding end-of-life issues. They sought attentive listening, recognition of their individual needs and honest conversations about their health. | Quest story | 10/10 |
| Lennaerts-Kats et al. J Parkinsons Dis. 2020; 10(4): 1631–1642. (6) | Qualitative semi-structured interviews |  | 10 | To document the experiences of family caregivers during both the palliative care phase and after the death of their loved one with PD. | NA | Individuals with PD believed carers relied on ad-hoc approaches rather than proactive ones, which could have offered a clearer future. Timely discussions were lacking. | When individuals with PD couldn’t speak for themselves, caregivers found it stressful to make treatment decisions, sometimes conflicting with the patient's preferences. | Caregivers highlighted the need for healthcare professionals to recognize the dependency of individuals with advanced PD on others for daily activities. They noted communication challenges due to apathy or cognitive impairment, especially in nursing home or hospital settings. | Chaos story | 10/10 |
| Prizer et al. J Appl Gerontol. 2020 Aug;39(8):834-845. (7) | Mixed-methods study (qualitative interviews complement quantitative survey findings. | 50 | 50 neurologists | To pinpoint the palliative needs of PD patients, explore the connection between palliative needs and HRQoL and investigate factors influencing HRQoL. | Individuals with PD received little guidance or educational support from their neurologists to manage their care, with many not recalling receiving such information. | NA | The study highlights the vital role of support groups in offering crucial education and referrals not typically provided by healthcare providers, especially in psychosocial support, aiding patients in coping with their illness. | Some participants doubted their physicians' communication with each other, while others felt responsible for conveying healthcare information among their providers. | Quest story | 10/10 |
| Klietz et al. Front Neurol. 2018 Mar 6:9:120.(8) | Structured interview and survey | 76 |  | To assess palliative care and quality of life in a local group of advanced PD patients to improve future care. | 72% of individuals with PD had a need for more information about palliative care, particularly regarding advance care planning for end-of-life care. | NA | 70% of individuals with PD had ADs and healthcare proxies. | Few received extra PC at home or consulted their treating physicians on palliative care issues, while nearly half of individuals with PD preferred discussing it with their GP or outpatient neurologist. | Chaos story (The PDQ-39 scale HRQOL correlated with MDS-UPDRS items ‘depressed mood and anxious mood’) | 10/10 |
| Badger et al. BMJ Support Palliat Care. 2018 Jun;8(2):180-183. (9) | Semi-structured interviews | 3 | 5 | Assess the impact of access to specialist palliative care services on the coping mechanisms of individuals with PD and their caregivers. | NA | NA | PC offered caregivers respite while allowing patients to rediscover themselves beyond their illness. | NA | Chaos story | 6/10 |
| Fox et al. Mov Disord Clin Pract. 2015 Apr 27;2(2):142-148. (10) | Survey with closed- and open-ended question |  | 306 | To survey Irish cargegivers knowledge, attitudes, and prior training on palliative care needs in PD | Many caregivers associated palliative care solely with end-of-life care, or care provided in the final days and hours. | NA | Many respondents stressed the importance of involving family members and informing them about "what to expect," as well as the necessity for education on the disease's nature, progression, and prognosis. | NA | NA | 10/10 |
| Walker et al. BMJ Support Palliat Care. 2014 Mar;4(1):64-7. (11) | Retrospective review of hospital notes and recorded data | 236 |  | To study adherence to palliative care guidelines for individuals with PD at end of life. | None of the individuals with PD had established an advanced care directive. | NA | NA | NA | NA | 10/12 |
| Hasson et al. Palliat Med. 2010 Oct;24(7):731-6. (12) | Qualitative semi-structured interviews |  | 15 | To investigate the lived experiences of former carers regarding palliative and end-of-life care. | Only a small number of caregivers were well-informed about PC, with a considerable portion perceiving them as intended for patients with cancer near EoL. | Accessing specialists led to lengthy waiting times for patients and caregivers. Interactions with specialists were often brief, focusing mainly on medication. | NA | Carers often turned to their GP for support, especially valuing home visits and information retrieval by GPs. However, some reported a lack of disease awareness among healthcare professionals, including GPs. | Chaos story | 9/10 |
| Giles et al. Palliat Med. 2009 Mar;23(2):120-5.(13) | A qualitative phenomenological conceptual framework | 3 | 4 | To assess the healthcare experiences for individuals and their families during the palliative stage of Parkinson's Disease? | Participants noted a lack of information on prognosis, diagnosis, and homecare services, unable to ask for what they needed. | NA | NA | Carers preferred hospital-based, multidisciplinary clinics for affordable, efficient, and credible care. Additionally, they emphasized the importance of holistic care beyond the disease itself. | Chaos story | 8/10 |
| Hudson et al. Palliat Med. 2006 Mar;20(2):87-94. (14) | Qualitative study | 8 | 21 | To depict the PD’s experience and explore the significance of palliative care. | NA | NA | Some caregivers discovered that the diminished interaction with the individual with PD was worsened by insufficient familial support. | PD sufferers and families who reported difficulties in 'staying connected' expressed feelings of psychological and social isolation. | Chaos story | 8/10 |
| Kundrick et al. Clin Park Relat Disord. 2023 Jun 20:9:100206. (15) | Cross-sectional survey study | 106 | NA | To study how individuals with PD perceive palliative care. | One-third of patients (n= 36) stated they had never encountered palliative care, while an extra 25% had heard of it but were uncertain of its meaning. | 37.7% felt neurologists should discuss ACP early in their illness. | NA | 48.1% preferred their neurologist should introduce them to palliative care | NA | 9/10 |
| Fox et al. Palliat Med. 2017 Jul;31(7):634-641. (16) | Qualitative study using semi-structured interviews | 19 | 12 | To explore key palliative care issues for individuals with PD and families and examine perceptions of palliative care. | Several participants were unfamiliar with the term "palliative care," while others misconstrued it as 'home help' or 'counseling,' or believed it was exclusive to those with a cancer diagnosis. | Early-stage individuals with PD avoided thinking about the future, while those in advanced stages, with declining medication effectiveness, were more open to ACP | Caregivers faced challenges in making difficult decisions regarding the care of their loved ones and expressed a desire for support. | Limited contact with healthcare teams left some individuals with PD and caregivers feeling isolated, unsupported, and overlooked regarding their social and psychological needs. Physicians often focused only on medication needs, overlooking equally distressing social and psychological concerns. | Chaos story | 10/10 |
| Bock et al. J Pain Symptom Manage. 2022 Jun; 63(6): 1031–1040. (17) | Nonblinded, pragmatic RCT | 104 usual care  106 usual care + PC | 88 usual care  87 usual care + PC | To identify the most effective aspects of a palliative care intervention and discover key factors associated with improvements in outcome. | NA | NA | NA | Completing ADs showed a slight reduction in caregiver burden at 12 months for the overall group, but this significance was lost when examining only the intervention group. | NA | 8/11 |
| Karmur et al. *I. Journal of Current Pharm Review and Res 2025; 17(5); 346-350. (18)* | *Cross-sectional questionnaire study.* | 100 |  | To assess treatment preferences regarding end-of-life care among patients with Parkinson’s disease. | 42% had already discussed their end-of-life preferences with family or healthcare providers.  21% knew about advance directives or do not resuscitate (DNR) orders, but 58% wanted to create one after the interview. | NA | 49% preferred shared decisions with their doctor and family, 31% wanted to decide independently, and 20% left decisions entirely to family. | NA | NA | 9/10 |
| PD, parkinson’s disease; EOL, end of life care; ACP, advance care planning; RCT, randomized controlled trial; ADs, advance directive | | | | | | | | | | |

**References**

1. Bock MA, Macchi ZA, Harrison KL, Katz M, Dini M, Jones J, et al. Does a novel community-based outpatient palliative care intervention for Parkinson's disease and related disorders improve care? Qualitative results from patients and care partners. Palliat Med. 2024;38(2):240-50.

2. Seshadri S, Contento A, Sugiura K, Abendroth M, Macchi Z, Kluger BM. Parkinson's Disease Carepartners' Perceptions of the Challenges and Rewards of Caregiving. Am J Hosp Palliat Care. 2024:10499091231223739.

3. Seshadri S, Dini M, Corcoran J, Job A, Contento A, Norton SA, et al. Parkinson disease patients' and carepartners' perceptions of palliative care. Parkinsonism Relat Disord. 2024;119:105982.

4. Chen Y, Zhang R, Lou Y, Li W, Yang H. Facilitators and barriers to the delivery of palliative care to patients with Parkinson's disease: a qualitative study of the perceptions and experiences of stakeholders using the socio-ecological model. BMC Health Serv Res. 2023;23(1):215.

5. Lennaerts-Kats H, Ebenau A, van der Steen JT, Munneke M, Bloem BR, Vissers KCP, et al. "No One Can Tell Me How Parkinson's Disease Will Unfold": A Mixed Methods Case Study on Palliative Care for People with Parkinson's Disease and Their Family Caregivers. J Parkinsons Dis. 2022;12(1):207-19.

6. Lennaerts-Kats H, Ebenau A, Steppe M, van der Steen JT, Meinders MJ, Vissers K, et al. "How Long Can I Carry On?" The Need for Palliative Care in Parkinson's Disease: A Qualitative Study from the Perspective of Bereaved Family Caregivers. J Parkinsons Dis. 2020;10(4):1631-42.

7. Prizer LP, Gay JL, Wilson MG, Emerson KG, Glass AP, Miyasaki JM, et al. A Mixed-Methods Approach to Understanding the Palliative Needs of Parkinson's Patients. J Appl Gerontol. 2020;39(8):834-45.

8. Klietz M, Tulke A, Müschen LH, Paracka L, Schrader C, Dressler DW, et al. Impaired Quality of Life and Need for Palliative Care in a German Cohort of Advanced Parkinson's Disease Patients. Front Neurol. 2018;9:120.

9. Badger NJ, Frizelle D, Adams D, Johnson MJ. Impact of specialist palliative care on coping with Parkinson's disease: patients and carers. BMJ Support Palliat Care. 2018;8(2):180-3.

10. Fox S, Gannon E, Cashell A, Kernohan WG, Lynch M, McGlade C, et al. Survey of Health Care Workers Suggests Unmet Palliative Care Needs in Parkinson's Disease. Mov Disord Clin Pract. 2015;2(2):142-8.

11. Walker RW, Churm D, Dewhurst F, Samuel M, Ramsell A, Lawrie C, et al. Palliative care in people with idiopathic Parkinson's disease who die in hospital. BMJ Support Palliat Care. 2014;4(1):64-7.

12. Hasson F, Kernohan WG, McLaughlin M, Waldron M, McLaughlin D, Chambers H, et al. An exploration into the palliative and end-of-life experiences of carers of people with Parkinson's disease. Palliat Med. 2010;24(7):731-6.

13. Giles S, Miyasaki J. Palliative stage Parkinson's disease: patient and family experiences of health-care services. Palliat Med. 2009;23(2):120-5.

14. Hudson PL, Toye C, Kristjanson LJ. Would people with Parkinson's disease benefit from palliative care? Palliat Med. 2006;20(2):87-94.

15. Kundrick A, Hogue O, Namrow S, Samala R, Walter E, Walter B, et al. Adopting a palliative care mindset is an unmet need in Parkinson's disease. Clin Park Relat Disord. 2023;9:100206.

16. Fox S, Cashell A, Kernohan WG, Lynch M, McGlade C, O'Brien T, et al. Palliative care for Parkinson's disease: Patient and carer's perspectives explored through qualitative interview. Palliat Med. 2017;31(7):634-41.

17. Bock M, Katz M, Sillau S, Adjepong K, Yaffe K, Ayele R, et al. What's in the Sauce? The Specific Benefits of Palliative Care for Parkinson's Disease. J Pain Symptom Manage. 2022;63(6):1031-40.

18. Karmur AM, Sipai RU, Mori DA, Gojiya AV. Treatment Preferences at the End of Life in Parkinson's Disease Patients: A Study at GMERS Medical College and Hospital, Junagadh. International Journal of Current Pharmaceutical Review and Research. 2025;17(5):346-50.
