# Supplementary material for: Patient autonomy in advance care planning for Parkinson’s disease: Systematic review with narrative synthesis
Source: Palliat Support Care. 2026 Jul 23;24:e204. doi: 10.1017/S1478951526103277 (PMC13430485; doi:10.1017/S1478951526103277)
Supplement: Bouma et al. supplementary material [file S1478951526103277sup001.zip › S1478951526103277sup001/Supplemental File 1 Search Strategy.doc]

Databases:

**PubMed**

<http://www.ncbi.nlm.nih.gov/pubmed?otool=leiden>

(("Parkinson Disease"[Mesh] OR "Parkinson Disease"[tw] OR "Parkinsonian Disorders"[Mesh] OR "Lewy Body Disease"[tw] OR "MPTP Poisoning"[tw] OR "parkinson*"[tw] OR "parkinson*"[ad]) AND ("Advance Care Planning"[Mesh] OR "Advance care planning"[tw] OR "Advanced care planning"[tw] OR "Advance care plan*"[tw] OR "Advanced care plan*"[tw] OR "Advance Medical Planning"[tw] OR "Advance Directive"[tw] OR "Advance Directives"[tw] OR "Living Will"[tw] OR "Living Wills"[tw] OR "Advance care planning"[title/abstract:~6] OR "Advance Medical Planning"[title/abstract:~6] OR "Advance Healthcare Directive"[tw] OR "Advance Healthcare Directives"[tw] OR "Critical Care Plan*"[tw] OR "End-of-Life Decision Making"[tw] OR "End-of-Life Plan*"[tw] OR "Future Care Plan*"[tw] OR "Healthcare Directive"[tw] OR "Healthcare Directives"[tw] OR "Healthcare Proxies"[tw] OR "Healthcare Proxy"[tw] OR "Life Care Planning"[tw]))

**Embase**

<http://ovidsp.ovid.com/ovidweb.cgi?T=JS&PAGE=main&MODE=ovid&D=oemezd>

((exp *"Parkinson Disease"/ OR "Parkinson Disease".ti,ab OR exp *"Parkinsonism"/ OR "Lewy Body Disease".ti,ab OR "MPTP Poisoning".ti,ab OR "parkinson*".ti,ab OR "parkinson*".in) AND (exp *"Advance Care Planning"/ OR "Advance care planning".ti,ab OR "Advanced care planning".ti,ab OR "Advance care plan*".ti,ab OR "Advanced care plan*".ti,ab OR "Advance Medical Planning".ti,ab OR "Advance Directive".ti,ab OR "Advance Directives".ti,ab OR "Living Will".ti,ab OR "Living Wills".ti,ab OR "Advance Healthcare Directive".ti,ab OR "Advance Healthcare Directives".ti,ab OR "Critical Care Plan*".ti,ab OR "End-of-Life Decision Making".ti,ab OR "End-of-Life Plan*".ti,ab OR "Future Care Plan*".ti,ab OR "Healthcare Directive".ti,ab OR "Healthcare Directives".ti,ab OR "Healthcare Proxies".ti,ab OR "Healthcare Proxy".ti,ab OR "Life Care Planning".ti,ab)) NOT (conference review or conference abstract).pt

**Web of Science**

<http://isiknowledge.com/wos>

((TI=("Parkinson Disease" OR "Parkinson Disease" OR "Parkinsonism" OR "Lewy Body Disease" OR "MPTP Poisoning" OR "parkinson*") OR AK=("Parkinson Disease" OR "Parkinson Disease" OR "Parkinsonism" OR "Lewy Body Disease" OR "MPTP Poisoning" OR "parkinson*") OR AB=("Parkinson Disease" OR "Parkinson Disease" OR "Parkinsonism" OR "Lewy Body Disease" OR "MPTP Poisoning" OR "parkinson*")) AND (TI=("Advance Care Planning" OR "Advance care planning" OR "Advanced care planning" OR "Advance care plan*" OR "Advanced care plan*" OR "Advance Medical Planning" OR "Advance Directive" OR "Advance Directives" OR "Living Will" OR "Living Wills" OR "Advance Healthcare Directive" OR "Advance Healthcare Directives" OR "Critical Care Plan*" OR "End-of-Life Decision Making" OR "End-of-Life Plan*" OR "Future Care Plan*" OR "Healthcare Directive" OR "Healthcare Directives" OR "Healthcare Proxies" OR "Healthcare Proxy" OR "Life Care Planning") OR AK=("Advance Care Planning" OR "Advance care planning" OR "Advanced care planning" OR "Advance care plan*" OR "Advanced care plan*" OR "Advance Medical Planning" OR "Advance Directive" OR "Advance Directives" OR "Living Will" OR "Living Wills" OR "Advance Healthcare Directive" OR "Advance Healthcare Directives" OR "Critical Care Plan*" OR "End-of-Life Decision Making" OR "End-of-Life Plan*" OR "Future Care Plan*" OR "Healthcare Directive" OR "Healthcare Directives" OR "Healthcare Proxies" OR "Healthcare Proxy" OR "Life Care Planning") OR AB=("Advance Care Planning" OR "Advance care planning" OR "Advanced care planning" OR "Advance care plan*" OR "Advanced care plan*" OR "Advance Medical Planning" OR "Advance Directive" OR "Advance Directives" OR "Living Will" OR "Living Wills" OR "Advance Healthcare Directive" OR "Advance Healthcare Directives" OR "Critical Care Plan*" OR "End-of-Life Decision Making" OR "End-of-Life Plan*" OR "Future Care Plan*" OR "Healthcare Directive" OR "Healthcare Directives" OR "Healthcare Proxies" OR "Healthcare Proxy" OR "Life Care Planning"))) NOT DT=(meeting abstract)

**Cochrane**

<https://www.cochranelibrary.com/advanced-search/search-manager>

(("Parkinson Disease" OR "Parkinson Disease" OR "Parkinsonism" OR "Lewy Body Disease" OR "MPTP Poisoning" OR "parkinson*") AND ("Advance Care Planning" OR "Advance care planning" OR "Advanced care planning" OR "Advance care plan*" OR "Advanced care plan*" OR "Advance Medical Planning" OR "Advance Directive" OR "Advance Directives" OR "Living Will" OR "Living Wills" OR "Advance Healthcare Directive" OR "Advance Healthcare Directives" OR "Critical Care Plan*" OR "End-of-Life Decision Making" OR "End-of-Life Plan*" OR "Future Care Plan*" OR "Healthcare Directive" OR "Healthcare Directives" OR "Healthcare Proxies" OR "Healthcare Proxy" OR "Life Care Planning")):ti,ab,kw

**CINAHL**

<https://catalogue.leidenuniv.nl/view/action/uresolver.do?operation=resolveService&package_service_id=33137965460002711&institutionId=2711&customerId=2710&VE=true>

Limit to Academic Journals

((TI("Parkinson Disease" OR "Parkinson Disease" OR "Parkinsonism" OR "Lewy Body Disease" OR "MPTP Poisoning" OR "parkinson*") OR SU("Parkinson Disease" OR "Parkinson Disease" OR "Parkinsonism" OR "Lewy Body Disease" OR "MPTP Poisoning" OR "parkinson*") OR MH("Parkinson Disease" OR "Parkinson Disease" OR "Parkinsonism" OR "Lewy Body Disease" OR "MPTP Poisoning" OR "parkinson*") OR MW("Parkinson Disease" OR "Parkinson Disease" OR "Parkinsonism" OR "Lewy Body Disease" OR "MPTP Poisoning" OR "parkinson*") OR AB("Parkinson Disease" OR "Parkinson Disease" OR "Parkinsonism" OR "Lewy Body Disease" OR "MPTP Poisoning" OR "parkinson*")) AND (TI("Advance Care Planning" OR "Advance care planning" OR "Advanced care planning" OR "Advance care plan*" OR "Advanced care plan*" OR "Advance Medical Planning" OR "Advance Directive" OR "Advance Directives" OR "Living Will" OR "Living Wills" OR "Advance Healthcare Directive" OR "Advance Healthcare Directives" OR "Critical Care Plan*" OR "End-of-Life Decision Making" OR "End-of-Life Plan*" OR "Future Care Plan*" OR "Healthcare Directive" OR "Healthcare Directives" OR "Healthcare Proxies" OR "Healthcare Proxy" OR "Life Care Planning") OR SU("Advance Care Planning" OR "Advance care planning" OR "Advanced care planning" OR "Advance care plan*" OR "Advanced care plan*" OR "Advance Medical Planning" OR "Advance Directive" OR "Advance Directives" OR "Living Will" OR "Living Wills" OR "Advance Healthcare Directive" OR "Advance Healthcare Directives" OR "Critical Care Plan*" OR "End-of-Life Decision Making" OR "End-of-Life Plan*" OR "Future Care Plan*" OR "Healthcare Directive" OR "Healthcare Directives" OR "Healthcare Proxies" OR "Healthcare Proxy" OR "Life Care Planning") OR MH("Advance Care Planning" OR "Advance care planning" OR "Advanced care planning" OR "Advance care plan*" OR "Advanced care plan*" OR "Advance Medical Planning" OR "Advance Directive" OR "Advance Directives" OR "Living Will" OR "Living Wills" OR "Advance Healthcare Directive" OR "Advance Healthcare Directives" OR "Critical Care Plan*" OR "End-of-Life Decision Making" OR "End-of-Life Plan*" OR "Future Care Plan*" OR "Healthcare Directive" OR "Healthcare Directives" OR "Healthcare Proxies" OR "Healthcare Proxy" OR "Life Care Planning") OR MW("Advance Care Planning" OR "Advance care planning" OR "Advanced care planning" OR "Advance care plan*" OR "Advanced care plan*" OR "Advance Medical Planning" OR "Advance Directive" OR "Advance Directives" OR "Living Will" OR "Living Wills" OR "Advance Healthcare Directive" OR "Advance Healthcare Directives" OR "Critical Care Plan*" OR "End-of-Life Decision Making" OR "End-of-Life Plan*" OR "Future Care Plan*" OR "Healthcare Directive" OR "Healthcare Directives" OR "Healthcare Proxies" OR "Healthcare Proxy" OR "Life Care Planning") OR AB("Advance Care Planning" OR "Advance care planning" OR "Advanced care planning" OR "Advance care plan*" OR "Advanced care plan*" OR "Advance Medical Planning" OR "Advance Directive" OR "Advance Directives" OR "Living Will" OR "Living Wills" OR "Advance Healthcare Directive" OR "Advance Healthcare Directives" OR "Critical Care Plan*" OR "End-of-Life Decision Making" OR "End-of-Life Plan*" OR "Future Care Plan*" OR "Healthcare Directive" OR "Healthcare Directives" OR "Healthcare Proxies" OR "Healthcare Proxy" OR "Life Care Planning")))

**PsycINFO**

<http://search.ebscohost.com/login.aspx?authtype=ip,uid&profile=lumc&defaultdb=psyh>

((TI("Parkinson Disease" OR "Parkinson Disease" OR "Parkinsonism" OR "Lewy Body Disease" OR "MPTP Poisoning" OR "parkinson*") OR SU("Parkinson Disease" OR "Parkinson Disease" OR "Parkinsonism" OR "Lewy Body Disease" OR "MPTP Poisoning" OR "parkinson*") OR MA("Parkinson Disease" OR "Parkinson Disease" OR "Parkinsonism" OR "Lewy Body Disease" OR "MPTP Poisoning" OR "parkinson*") OR MJ("Parkinson Disease" OR "Parkinson Disease" OR "Parkinsonism" OR "Lewy Body Disease" OR "MPTP Poisoning" OR "parkinson*") OR AB("Parkinson Disease" OR "Parkinson Disease" OR "Parkinsonism" OR "Lewy Body Disease" OR "MPTP Poisoning" OR "parkinson*")) AND (TI("Advance Care Planning" OR "Advance care planning" OR "Advanced care planning" OR "Advance care plan*" OR "Advanced care plan*" OR "Advance Medical Planning" OR "Advance Directive" OR "Advance Directives" OR "Living Will" OR "Living Wills" OR "Advance Healthcare Directive" OR "Advance Healthcare Directives" OR "Critical Care Plan*" OR "End-of-Life Decision Making" OR "End-of-Life Plan*" OR "Future Care Plan*" OR "Healthcare Directive" OR "Healthcare Directives" OR "Healthcare Proxies" OR "Healthcare Proxy" OR "Life Care Planning") OR SU("Advance Care Planning" OR "Advance care planning" OR "Advanced care planning" OR "Advance care plan*" OR "Advanced care plan*" OR "Advance Medical Planning" OR "Advance Directive" OR "Advance Directives" OR "Living Will" OR "Living Wills" OR "Advance Healthcare Directive" OR "Advance Healthcare Directives" OR "Critical Care Plan*" OR "End-of-Life Decision Making" OR "End-of-Life Plan*" OR "Future Care Plan*" OR "Healthcare Directive" OR "Healthcare Directives" OR "Healthcare Proxies" OR "Healthcare Proxy" OR "Life Care Planning") OR MA("Advance Care Planning" OR "Advance care planning" OR "Advanced care planning" OR "Advance care plan*" OR "Advanced care plan*" OR "Advance Medical Planning" OR "Advance Directive" OR "Advance Directives" OR "Living Will" OR "Living Wills" OR "Advance Healthcare Directive" OR "Advance Healthcare Directives" OR "Critical Care Plan*" OR "End-of-Life Decision Making" OR "End-of-Life Plan*" OR "Future Care Plan*" OR "Healthcare Directive" OR "Healthcare Directives" OR "Healthcare Proxies" OR "Healthcare Proxy" OR "Life Care Planning") OR MJ("Advance Care Planning" OR "Advance care planning" OR "Advanced care planning" OR "Advance care plan*" OR "Advanced care plan*" OR "Advance Medical Planning" OR "Advance Directive" OR "Advance Directives" OR "Living Will" OR "Living Wills" OR "Advance Healthcare Directive" OR "Advance Healthcare Directives" OR "Critical Care Plan*" OR "End-of-Life Decision Making" OR "End-of-Life Plan*" OR "Future Care Plan*" OR "Healthcare Directive" OR "Healthcare Directives" OR "Healthcare Proxies" OR "Healthcare Proxy" OR "Life Care Planning") OR AB("Advance Care Planning" OR "Advance care planning" OR "Advanced care planning" OR "Advance care plan*" OR "Advanced care plan*" OR "Advance Medical Planning" OR "Advance Directive" OR "Advance Directives" OR "Living Will" OR "Living Wills" OR "Advance Healthcare Directive" OR "Advance Healthcare Directives" OR "Critical Care Plan*" OR "End-of-Life Decision Making" OR "End-of-Life Plan*" OR "Future Care Plan*" OR "Healthcare Directive" OR "Healthcare Directives" OR "Healthcare Proxies" OR "Healthcare Proxy" OR "Life Care Planning")))
